# Measuring What Matters: Using Participatory Choice Modelling to Understand Preferences for Homelessness Integrated Care

**DOI:** 10.5334/ijic.9846

**Published:** 2026-08-05

**Authors:** Michela Tinelli, Gopika Rajeendar, Michelle Cornes, Jess Harris, Raphael Wittenberg, Michael Clark

**Affiliations:** 1 Care Policy and Evaluation Centre at the London School of Economics and Political Science, United Kingdom; 2 Research and Innovation, London School of Economics and Political Science, United Kingdom; 3 University of Salford, United Kingdom; 4 Health and Social Care Workforce Research Unit, King’s College London, United Kingdom

**Keywords:** participatory research, discrete choice experiments, homelessness, preference elicitation, integrated care, hard-to-reach populations

## Abstract

**Introduction:**

People with experience of homelessness (PEH) often have multiple inter-related needs and for support rely on various services working together. To improve Out-of-Hospital Care (OOHC), it is crucial to understand their preferences. This study used a Discrete-Choice Experiment (DCE) to identify what matters most to them, ensuring services align with real-life experiences.

**Methods:**

A mixed methods approach combined interviews, group discussions, and DCE data. Service users, providers, and planners participated in workshops. Researchers with lived experience of homelessness co-designed a user-friendly questionnaire and supported data collection, fostering trust. Preferences were analysed using logistic regression, with findings presented in clear, interactive dashboards.

**Results:**

A total of 112 PEH participated from 10 sites, with 108 valid responses (35% response rate). Participants preferred care delivered at home, delivered by trusted housing workers, with minimal behavioural restrictions. Accommodation type and behavioural rules significantly influenced service uptake. DCE outputs were shared with PEH, who confirmed that the findings accurately reflected their priorities and real-life experiences. In one locality, insights were used to reshape service modelling.

**Discussion & conclusion:**

This study highlights the importance of co-designing OOHC services with PEH. Stability, trust, and fewer restrictions encourage service engagement. These insights can guide policymakers and service providers in developing more effective, person-centred care models, ultimately improving access and outcomes for PEH.

## Introduction

Economic evaluation research has evolved considerably. Once focused mainly on healthcare, it now spans social care and integrated care services—an important shift for addressing the needs of people who are often overlooked, such as those with experience of homelessness (PEH) and multiple disadvantage (defined as overlapping experiences of homelessness, substance use, mental health issues, and justice involvement) [1,2]. Understanding what matters most to these individuals is challenging because of their complex circumstances. Traditional research methods frequently fail to capture their priorities, leaving gaps in how services respond to real-world needs [3,4].

Homelessness, particularly multiple exclusion homelessness, is a deeply complex issue with significant social and economic consequences [5]. It is linked to poorer health outcomes, premature mortality, increased use of emergency healthcare, greater involvement with the justice system, and reduced work productivity [2,6]. People experiencing homelessness (PEH) face profound health inequities driven by social determinants such as poverty, unstable or absence of housing, and limited access to coordinated care. This can lead to delays, gaps, and missed opportunities for recovery [7,8].

Integrated care provides a framework for addressing these challenges by promoting person-centred, joined-up services across health, housing, and social support. The International Foundation for Integrated Care (IFIC) identifies nine pillars of integration, including, for example, “people as partners in care,” “population health management,” and “co-ordinated care”, all critical for improving outcomes for PEH [9,10]. Within this context, Out-of-Hospital Care (OOHC) models, such as step-down care, play a pivotal role in reducing hospital admissions and supporting recovery in community settings. However, designing OOHC services that reflect the preferences of PEH remains a challenge, particularly given the complexity of multiple exclusion homelessness. Understanding these preferences is essential for creating equitable, person-centred care pathways.

Discrete Choice Experiments (DCEs) [11,12,13,14] are widely used in health and care economics to generate data that can be analysed using choice modelling to quantify trade-offs between service attributes. However, their application with marginalised populations remains limited [15]. This study addresses that gap by employing a participatory DCE approach, co-designed with individuals with lived experience of homelessness. Building on evidence of successful co-design in mental health and cancer prevention initiatives [16,17], our approach engaged stakeholders from attribute development through data collection, analysis, visualisation and insights interpretation. By incorporating non-clinical features such as trust in providers and flexibility in behavioural rules (often overlooked in conventional service design) this study provides actionable insights for integrated OOHC models. Findings are operationalised through an interactive dashboard to support service providers and commissioners in implementing person-centred, integrated care solutions. Given the relevance of choice modelling to designing person-centred services, this study uses a participatory DCE survey approach—details of the rationale, design, and implementation are provided in the Methods section. By involving stakeholders from the outset, including selecting attributes, designing surveys, and interpreting results, participatory DCEs ensure research reflects real needs and priorities [13,18]. Conducting DCEs with vulnerable groups, however, brings ethical and practical challenges, such as obtaining informed consent, protecting participants from distress, and navigating power dynamics [19,20,21,22,23].

This study builds on these principles and is among the first to apply a participatory DCE approach with PEH to inform integrated OOHC design. Its originality lies in engaging individuals with lived experience throughout (from attribute development to data collection and interpretation), while incorporating non-clinical features such as behavioural rules and trust in care providers, which are often overlooked in conventional service planning. Findings were operationalised through an interactive dashboard, offering commissioners and policymakers a practical tool for designing person-centred, integrated care models. We present findings from a case study involving a DCE survey conducted as part of a broader programme assessing OOHC models in England [24]. The paper also offers recommendations for future research, highlighting the potential of participatory DCEs to inform more effective and sustainable care models for marginalised populations [25,26,27].

## Research methods

This study is part of a larger evaluation programme examining OOHC models for PEH in England [24]. This specific case study explores preferences for different OOHC service configurations among PEH. The overarching programme’s goal is to develop and put into practice better OOHC models, ultimately improving individual outcomes and preventing people from returning to homelessness.

To achieve the study objectives, we adopted a mixed-methods approach, combining quantitative data obtained through a structured DCE questionnaire, analysed using choice modelling techniques, with qualitative insights from stakeholder consultations.

### Quantitative data

Choice modelling was selected as the primary preference elicitation method as it enables a structured assessment of trade-offs between multiple service attributes, which is essential for designing integrated care models. DCEs are widely employed in health economics and integrated care research to quantify preferences by presenting respondents with hypothetical scenarios that vary across key attributes [11,12,13,14]. This approach allows estimation of the relative importance of service features and the trade-offs individuals are willing to make, providing actionable insights for resource allocation and service design.

When completing a DCE questionnaire, people are asked to choose between different service options. For example, they might compare two hospital discharge plans that differ in where care is provided, who delivers it, how long it lasts, the type of accommodation offered, and the level of support available. These choices help us understand what matters most to them.

DCEs were particularly suited to this study because they:

- Capture preferences across several aspects of care, such as location, provider type, behavioural expectations, and accommodation.
- Show the trade-offs people are willing to make, for example, accepting a short delay in care if it means receiving support at home rather than in hospital [28]. This insight is vital for planning services and allocating resources effectively.
- Predict how likely people are to use different service configurations, helping commissioners make evidence-based decisions.
- Put people at the centre by revealing their priorities, rather than relying on assumptions.

### Qualitative data

The qualitative section involved workshops and webinars with a variety of stakeholders, including service providers, local commissioners, central government, public health authorities, homelessness charities, and individuals with personal/lived experience of homelessness. This group involvement is referred to as PPPIE – People, Practitioner, Policy Involvement, and Engagement stakeholders.

The step-by-step participatory table illustrates how stakeholders were engaged at each stage of the DCE process, ensuring transparency and demonstrating the depth of involvement from attribute development to interpretation (Table 1). The quantitative part focused on the DCE survey to understand preferences by presenting respondents with different hypothetical choices for services. By integrating both qualitative and quantitative methods, the study achieved a comprehensive understanding, combining real-life experiences with thorough statistical analysis. This, in turn, aids and informs the development of OOHC models that are more tailored to the needs of individuals. The study followed a participatory framework, based on principles such as collaboration, shared decision-making, and empowerment [29,30,31]. Involvement from diverse stakeholders was ensured at every stage to guarantee relevance, meaningful engagement, and practical impact. Acknowledging the importance of varied perspectives in research on vulnerable populations [32], the input from PPPIE stakeholders at each DCE stage is detailed below, in line with established DCE guidance [33]. The presentation of methods and findings was guided by a published checklist [34].

**Table 1:** DCE stages, critical steps we experienced, and how participatory research helped us address them.


DCE PHASE (REED JOHNSON ET AL., 2013)	CRITICAL STEPS WE EXPERIENCED	HOW PARTICIPATORY RESEARCH HELPED US ADDRESS THEM

**1. Attribute & level development**	Identify relevant attributes and define realistic levels.	Previous OOHC research informed the initial selection. Workshops/webinars with service providers (Local Government Association, LGA, charities). Key: PPI stakeholder consultation on attributes and levels.

**2. Questionnaire design**	Develop a clear, accessible questionnaire; ensure understandable choice tasks.	PPI stakeholder input on presentation, wording, and format. PPI review of drafts, feedback on clarity, and layout. Cognitive interviewing. Pilot testing (n = 88).

**3. Data collection**	Recruit a representative sample; administer effectively; provide support.	Individuals with lived experience recruited from 10 sites (n = 300 enrolled, 112 collected and 108 suitable for analyses). Trained peer researchers supported respondents. Paper questionnaires.

**4. Data analysis & interpretation**	Analyse data; interpret results; and translate findings into recommendations.	PPPIE stakeholder involvement in interpretation. PEH consultation on translating findings into recommendations.

**5. Presentation & insights derivation**	Effectively communicate findings; derive actionable insights.	Interactive dashboards (co-created with all PPPIE stakeholders). Videos and website for communication. Workshops to present findings, demonstrate visualisations, and inform decision-making. Iterative dashboard refinement.


### Attribute and level development

To design our study, we first needed to identify the most important features of OOHC for PEH. In a DCE, people are asked to choose between different options based on a set of characteristics—called *attributes*—that matter to them. Each attribute has a range of possible *levels*, which represent different ways that feature might look in practice (for example, how quickly support is offered, or who provides the care). We began selecting the key attributes, or characteristics, and their levels for the DCE by looking at previous research focused on OOHC for PEH. After this initial selection, we held several workshops and online meetings to verify and refine these choices. These events brought together service providers in the Local Government Association (LGA) network, local commissioners, central government, public health authorities, and homelessness charities. In total, there were two events involving approximately 40 participants and three events with 50 participants who had direct experience with homelessness. The purpose of these sessions was to discuss what stakeholders valued in specialised services and to ensure that the attributes chosen matched real-world service delivery and the views of those involved in the sector. We asked PPPIE stakeholders specifically to share what aspects of OOHC were most important to them and which options would be practical and relevant. For example, discussions with PEH highlighted the importance of including “rules for behaviour in their living environment” as an attribute. These rules were seen as having a significant impact on their well-being and housing stability. This continuous process aimed to ensure that the DCE included attributes and levels that were meaningful and relevant to the target population, thereby improving the content validity of the tool [35]. The final list of attributes and levels we arrived at included:

- **Location of care:** options such as a hospital, own home/flat, shared flat/house, hostel, hotel, or care home;
- **Professional providing care:** choices between a social worker, housing support worker, peer navigator, mental health support worker, or drug and alcohol support worker;
- **Frequency of care:** attendance could be 1-2 times, 3-4 times, 5-6 times, or 7 or more times per week;
- **Duration of care after leaving the hospital:** periods of 4-5 weeks, 6-7 weeks, 8-9 weeks, or 10-12 weeks;
- **Rules for behaviour in their living environment:** these could range from no rules to less strict, strict, or very strict rules.

### Questionnaire design

**Figure 1:**
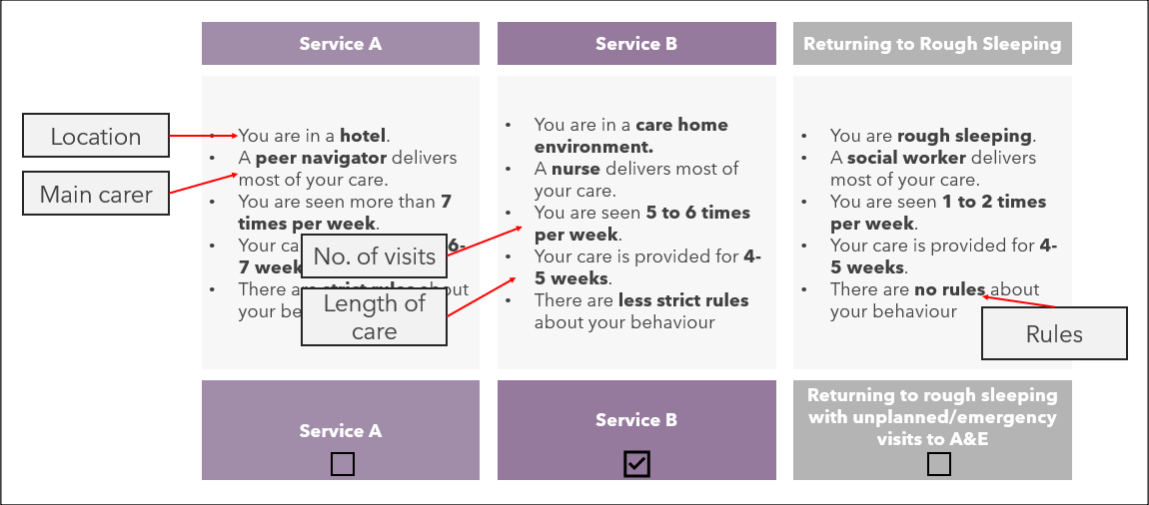
Example of DCE choice. Note: the list of service characteristics (attributes) and corresponding values (or levels) is presented in Appendix 1.

In the creation of the questionnaire, PPPIE stakeholders played a crucial role. They assisted in shaping the appearance, language, and format of the DCE questionnaire. This cooperative method sought to guarantee that the questionnaire was straightforward and understandable for PEH, taking into account any potential literacy issues and cognitive challenges [36]. PEH examined early versions of the questionnaire. They offered suggestions on the clarity of the language, the visual arrangement, and the overall usability. They also proposed ways to make the tasks simpler and less mentally demanding. For example, they recommended using straightforward, plain language and adding visual aids. To further enhance the questionnaire and ensure comprehension, cognitive interviews were conducted with four individuals who had experienced homelessness [37]. Cognitive interviews involve asking people to talk through their thought process as they answer each question (e.g., what they think the question means, how they arrive at their answer, and whether anything is confusing or unclear). This method helps researchers identify any wording or concepts that may be misunderstood, so we can improve the questionnaire and make sure it reflects the experiences and perspectives of the people it’s designed for. A trial run with a small group of 10 people with similar experiences helped identify any remaining problems with the questionnaire, how the data were collected, and the econometric model. Advanced experimental design techniques [38] were used to develop the DCE questions (Figure 1), reducing the cognitive load for respondents while gathering maximum information. Participants had to choose between two proposed service options (Service A or B), each with different combinations of the five care model attributes, compared with a fixed rough sleeping scenario. Each participant completed five choice scenarios. Further details are provided in Appendices 1–2.

### Data collection

The DCE was conducted with PEH recruited from 10 sites in the OOHCM evaluation [24], specifically before they left a Pathway 1 (Take home and ‘settle-in’ support defined as Reablement) or Pathway 2 (specialist bedded intermediate care and step-down houses) service. Across the 10 reporting sites, service models varied substantially, with four delivering Pathway 1 settle-in/reablement offers and six delivering Pathway 2 step-down/bedded models, reflecting different local strategies for post-discharge support and capacity. The scale and investment also differed, for example, from dispersed Pathway 1 units and floating support to larger, staffed step-down provisions, indicating variability in local needs, resource levels, staffing configurations, and reliance on multidisciplinary in-reach versus dedicated accommodation. For details on the diversity of the 10 sites and variability of the cohorts, please refer to [24]. A total of 300 individuals were invited to enrol in the study. This sample size was determined *a priori* using a rule of thumb calculation [39] to ensure sufficient statistical power for the analysis, taking into account the number of attributes, levels, and choice tasks. The calculation considered the largest number of levels for any one attribute (7), the number of choice tasks per respondent (5), and the number of alternatives per choice task (2). Recognising the potential cognitive load and the needs of the target population, trained peer researchers, who themselves had lived experience of homelessness, supported respondents to complete the questionnaire. This aimed to maximise response rates and ensure diverse perspectives, fostering trust and rapport [40]. Two peer researchers received training on the study protocol, the questionnaire, and ethical considerations. Two additional peer researchers and frontline staff at participating sites administered the paper questionnaires. Data were collected only from individuals providing written informed consent.

### Data analysis

Logit regression techniques [38] were used to analyse the DCE data, examining valued attributes, their relative importance, and preferred levels. The direction of preferences was assessed using variable coefficients. P-values (p < 0.10) determined attribute statistical significance. Coefficient size indicated relative importance. Subgroup analyses were conducted to explore the impact of patient characteristics (age, gender, location, and experience of care) on preferences. Regression outputs calculated probabilities of choosing specific service configurations versus returning to rough sleeping. These probabilities indicated expected uptake rates of different specialist hospital discharge schemes. More details on the analysis and correspond regression outputs are presented elsewhere (appendices 2 and 3, respectively).

### Presentation & insights derivation

A multi-faceted dissemination strategy ensured research findings reached and were adopted by diverse stakeholders. Interactive dashboards, providing visually accessible summaries of DCE results, were co-created with PPPIE stakeholders through five iterative workshops (with about 10–45 stakeholders in each). These dashboards enable data exploration, understanding of trade-off, and impact simulation of different service configurations. The stakeholders were consulted on how the findings could be translated into practical recommendations for service design and delivery. Experts with lived experience (4) were involved in the interpretation of the results, providing context and ensuring that the findings were meaningful and relevant to their lived experiences [18]. In addition to the co-created interactive dashboards, dissemination methods included videos, a dedicated website (www.qualityevaluation.com), and workshops to present findings, demonstrate visualisations, and inform decision-making (10 events, with about 250 people in total). The dashboards were iteratively refined based on stakeholder feedback on their needs. A sample of the visualisations are provided in appendix 4; demonstration of the interactive automated dashboarding prototype under development at the LSE is available upon request (MT).

### Ethical considerations

Ethical considerations were very important for this study. Since the study involved people who might be vulnerable, we made sure they understood every aspect of participation clearly. Ethical safeguards included clear, accessible information sheets, verbal explanations, and iterative consent checks throughout participation. We provided them with information about the study’s goals, what they would have to do, and any risks or benefits involved. Peer researchers with lived experience supported recruitment and data collection, fostering trust by creating a safe and relatable environment that helped participants feel understood and respected, and fostering autonomy by ensuring participants could make informed, voluntary decisions through clear communication, ongoing consent checks, and support that enabled them to stay in control of how they engaged with the study. Materials were designed to be user-friendly, and participants were reminded of their right to withdraw at any stage without facing any negative consequences. We also made sure that they understood how their information would be used and protected. To ensure their privacy, we anonymised the data and stored it securely, following data protection laws . The study was reviewed and approved by the Health Research Authority Social Care Research Ethics Committee [22/IEC08/0016].

## Results

### DCE responses

The DCE survey was well-received by study participants, with 108 completed questionnaires deemed suitable for analysis. This represented a 35% response rate from the total cohort of 300 individuals enrolled from the 10 participating sites between October 2022 and July 2023. The respondent sample was predominantly male (69%) with an average age of 49. Most respondents had experienced Pathway 1 care (56%) compared to Pathway 2 care (44%). See Table 2.

**Table 2:** Respondents’ characteristics (n = 108).


	MEAN	SD

**Age**	49	12

	**%**	**Number**

**Age categories**		

20–24	5	5

25–29	6	6

30–34	5	5

35–39	12	13

40–44	13	14

45–49	12	13

50–54	12	13

55–59	14	15

60–64	15	16

65–69	4	4

70–74	1	1

75–80	1	1

Gender*		

Male	69	73

Female	31	35

**Service received**		

Pathway 1	56	60

Pathway 2	44	48


Note: Pathway 1 refers to “Take home and settle-in” support, commonly defined as *Reablement*, a short-term intervention aiming to help individuals regain independence at home following a hospital stay. Pathway 2 involves access to *specialist bedded intermediate care* or *step-down housing*, providing structured support for those who are not yet able to return home safely but do not require acute hospital care. *Please note that nobody identified their gender in any other way.

### Presentation of DCE findings

Key insights from the DCE data analysis are outlined below.

**Figure 2:**
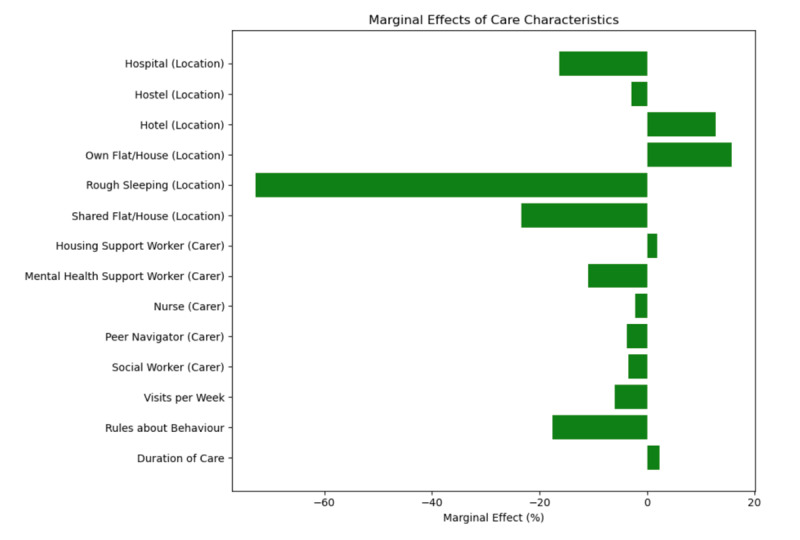
Relative importance of intermediate care characteristics. Note: This figure displays the marginal effects (%) of different intermediate care characteristics on individuals’ choices, derived from a DCE. These marginal effects quantify the relative importance of each attribute when PEH select a model of OOHC. For example, when looking at location: *Rough Sleeping* has the strongest negative marginal effect (~ –60%), indicating a very strong preference against this option; *Own Flat/House* is the most preferred location, showing the highest positive marginal effect (~ +20%).

*Main analysis (appendix 3)*: A strong aversion to returning to rough sleeping was evident, reinforcing qualitative findings about the high acceptability of step-down care (Cornes et al, 2024). All five attributes included in the DCE (location, professional providing care, frequency of care, duration of care, and rules for behaviour) significantly influenced individuals’ choices regarding specialist intermediate care. The relative importance of these attributes, ranked from most to least preferred, was: professional providing care, no rules about behaviour, location of care, duration of care (in weeks), and frequency of care (visits per week). The preferred service model, derived from the analysis, offered accommodation in one’s own flat/house, care provided by a housing support worker, no rules about behaviour, a duration of care of 10–12 weeks, and a frequency of care of 3–4 times per week (Figure 2).

*Subgroup analysis (appendix 4)*: Choice frequency for each attribute characteristic was analysed for the entire cohort, as well as for subgroups based on age, gender, location, and type of service received. Chi-Square test confirmed that there was no association between the participants choices and their age, gender, location, or pathway of care.

*Service uptake modelling (Appendix 4)*: Three illustrative examples demonstrate the impact of different service configurations on uptake probabilities compared to the baseline scenario of rough sleeping. These examples highlight the trade-offs individuals are willing to make.

- **Example 1: focus on accommodation:** Changing only the “location” attribute from “rough sleeping” to “own flat/house,” while holding all other attributes constant, dramatically increased the probability of service uptake from 13.5% to 86.5%. This underscores the strong motivating factor of securing independent accommodation (73%-point increase in the probability of choosing the target scenario compared to the baseline).
- **Example 2: focus on rules:** Even a small change in the “rules about behaviour” attribute, from “no rules” to “some rules,” significantly reduces the likelihood of choosing the target scenario (only 1.4%), with the vast majority (98.6%) still preferring the baseline. This demonstrates how even minimal behavioural restrictions can create a strong disincentive.
- **Example 3: multi-attribute change:** A hypothetical “ideal” service, incorporating long-term accommodation (10–12 weeks) in one’s own home, multidisciplinary team support (3–4 times per week), and no behavioural restrictions, resulted in a 90.6% probability of uptake, compared to the 9.4% probability associated with rough sleeping. This highlights the combined positive impact of these preferred attributes.

### Insights from the participatory process

The participatory approach, integral to all stages of this research, provided valuable insights beyond the quantitative DCE findings. Engaging PPPIE stakeholders, particularly PEH, enriched the study in several ways.

*Relevance and construct validity*. Iterative consultations with PPPIE contributors ensured the research addressed priorities grounded in real-world experiences. For example, while the initial attribute set for the DCE did not include “rules about behaviour,” stakeholders highlighted its critical influence on service engagement and housing stability. Its inclusion enhanced the construct validity of the DCE. Similarly, discussions around the “type of professional providing care” revealed strong preferences for support workers with lived experience or a deep understanding of homelessness, reinforcing the salience of trust and relatability in service delivery.

*Questionnaire design and accessibility*. PPPIE contributors improved the accessibility and clarity of the DCE instrument. Feedback led to revisions in question wording, layout, and the use of plain language, with examples introduced to support comprehension and reduce cognitive burden. These adaptations helped ensure the instrument was appropriate for a population often underserved in research due to complex life circumstances.

*Data collection and rapport*. Peer researchers—trained individuals with lived experience—were involved in recruitment and data collection. Their involvement fostered trust, increased response rates, and contributed to the ethical conduct of research with a vulnerable population. Participants reported feeling more at ease sharing perspectives with those who shared similar life experiences, thereby supporting the collection of rich and authentic data.

*Interpretation and contextualisation*. Stakeholders also contributed to the interpretation of findings. For instance, while the DCE data suggested a preference for “no rules,” qualitative input clarified that the underlying issue was not an absence of structure, but a desire for autonomy, dignity, and fairness in service expectations. This layered understanding strengthened the practical applicability of the findings. Results were shared with PPPIE stakeholders during a dedicated feedback session. Stakeholders expressed strong support for prioritising trust and stability in service delivery, emphasising that these elements are critical for engagement. They also recommended simplifying behavioural rules to reduce barriers to participation and ensure services feel more inclusive and person-centred.

*Capacity building and empowerment*. Beyond the immediate research benefits, the participatory process contributed to capacity building among peer researchers and PPPIE contributors. Many reported increased confidence, a sense of empowerment, and interest in further involvement in research or service improvement activities [41].

*Pathway to impact*. Insights from the participatory process, in combination with DCE results, informed the design of an interactive dashboard (CQE platform: qualityevaluation.com) and a series of policy workshops. One local authority has already used the evidence to co-design a revised OOHC model, demonstrating how participatory economic research can directly inform service commissioning and improve care outcomes.

## Discussion

This study adds to the growing evidence base on integrated care by showing how service users’ preferences—particularly those with lived experience of homelessness—can shape more responsive and joined-up models of care. Using participatory choice modelling, we identified what matters most to individuals when accessing OOHC, demonstrating how integrated care planning can be strengthened by embedding service user priorities from the outset.

A clear finding was the strong preference for “step-down care”—support that enables a safe and sustainable transition back into the community rather than returning to rough sleeping. This model was seen as highly acceptable. Key preferences included having a housing support worker and avoiding strict behavioural rules, alongside practical factors such as location, duration, and frequency of visits. Participants described their ideal service as living in their own flat or house, receiving visits from a support worker three to four times weekly, having 10–12 weeks of care, and no behavioural restrictions. Visualisation of DCE data through the dashboard provided further insights into how these preferences can inform practical service redesign and implementation.

### Framing within IFIC’s 9 Pillars

Our findings resonate strongly with several of IFIC’s pillars:

- Person-centred care: Preferences for living in a home-like environment and minimising behavioural restrictions reflect a clear desire for autonomy and dignity, echoing the principle of care that starts with the person, not the system.
- Population health management: The emphasis on stable housing and support workers aligns with addressing social determinants of health—critical for improving outcomes among PEH.
- Shared decision-making: The participatory approach and co-creation of the dashboard exemplify collaborative design, ensuring that those most affected have a voice in shaping services.
- Workforce development: The strong preference for housing support workers underscores the need for investment in training and sustaining this workforce.
- Digital solutions: The dashboard represents innovation in using technology to model service configurations and predict uptake, supporting informed commissioning.
- Governance and accountability: Co-production and stakeholder engagement throughout the process strengthen transparency and trust, key components of integrated governance.

Other pillars, such as financing, organisational integration, and community engagement, are central to the policy recommendations, particularly around scaling step-down housing and embedding lived experience in service design.

### Comparison with existing literature

Our findings echo existing evidence on homelessness and integrated care. The preference for housing-led models reflects the principles of *Housing First*, first demonstrated by Tsemberis et al. [42] and reinforced by UK evaluations [43,44]. These studies consistently show that immediate access to stable housing improves health and social outcomes far more effectively than conditional approaches. This highlights housing as a fundamental determinant of health, not a reward for compliance.

Participants’ rejection of strict behavioural rules mirrors critiques that such measures perpetuate stigma and undermine recovery [45]. Our findings reinforce the argument that integrated care must go beyond structural coordination to embrace relational and rights-based approaches, ensuring dignity and autonomy. Similarly, the emphasis on trust and therapeutic relationships aligns with research on relational care for vulnerable groups [46,47].

Achieving a 35% response rate in a participatory DCE with people experiencing homelessness is notable. It demonstrates feasibility and adds to the growing body of work advocating inclusive research methods [14,23]. This is particularly encouraging given challenges such as mobility, lack of stable contact details, and mistrust of institutions [48]. Adaptations (e.g., simplified language, visual aids, and peer researcher involvement) reflect best practice and ethical guidance for research with marginalised groups [30]. These strategies improved comprehension and built trust, enabling more authentic engagement.

### Strengths, challenges and limitations

*Strengths*. The participatory research approach offered significant benefits. It ensured that the study focused on issues that mattered to the target population, thereby improving the relevance and validity of the research findings [49]. Engagement and buy-in from stakeholders were increased, fostering a sense of ownership in the research process [30]. This method empowered marginalised groups by giving them a voice in shaping services that impact their lives [50]. Importantly, it facilitated the practical application of research findings to real-world service design and commissioning [51]. Highlighting housing as a key preference factor aligns with studies demonstrating the essential role of stable accommodation in recovering from homelessness [52]. Trust in care providers is crucial, supporting research that underscores the importance of therapeutic relationships in services for vulnerable groups [53]. The use of participatory methods is increasingly recognised as best practice in research with marginalised groups, reflecting a growing appreciation for the importance of lived experience in shaping service design [54]. While cognitive demands can pose challenges for PEH, we mitigated these through simplified language, visual aids, and iterative testing with peer researchers to ensure comprehension and relevance. These adaptations were informed by best practice guidelines for conducting DCEs with vulnerable populations [14,23].

To translate these preferences into practical solutions, we co-created an integrated management dashboard (*Care Quality Evaluation*, qualityevaluation.com). This tool, reportedly the first of its kind, allows commissioners and providers to model different service configurations and predict uptake. Combined with earlier evidence on commissioning challenges [44], our findings suggest that housing-led “step-down” models offer a promising option for those with less intensive rehabilitation needs. These models provide a familiar, home-like setting, avoid unsuitable temporary accommodation, and enable consistent face-to-face support—addressing weaknesses in outreach-based approaches.

*Challenges*. Several challenges were encountered in implementing this participatory DCE. Recruiting and retaining PEH required significant effort and flexibility [45]. Due to difficulties in communication and understanding, which were linked to literacy levels and mental strain, we needed to design the questionnaire carefully and involve peer researchers for advice and support. These peer researchers played a crucial role in building trust with participants, leading to more open and truthful responses [52]. Power dynamics among stakeholders had to be carefully managed to ensure fair participation and that everyone’s voice was heard. Limited time and resources, often a reality in participatory research, necessitated meticulous planning and management. Ethical considerations, particularly regarding informed consent and confidentiality, were paramount throughout the research process and needed constant attention and careful management. This approach aligns with established guidelines for ethical conduct in qualitative research involving vulnerable populations [53].

*Limitations*. A few factors should be considered when interpreting findings and applying them to broader contexts. For example, potential hypothetical bias inherent in DCEs [23] and limited generalisability beyond the study sites. Also, limited resources meant we could not translate questionnaires, which restricted our ability to include views from less-represented ethnic groups who are not fluent in English. Additionally, the study faced constraints in time and funding, which may have limited the depth of engagement and the diversity of perspectives captured.

### Implications for policy and practice

The study findings suggest that policymakers and service providers should prioritise stable and appropriate accommodation as a foundation for effective OOHC. Investing in training and support for housing support workers is crucial to ensure they can provide the required practical and emotional assistance to PEH. Services must be designed to be flexible and responsive to individual needs, minimising unnecessary restrictions and promoting independence. Co-producing services with individuals who have lived experience should be central to service design and delivery. National housing and health strategies should focus on increasing the availability of affordable and appropriate accommodation and support options, like step-down housing and supported living arrangements.

Funding models for OOHC services should encourage integrated care that addresses both housing and support needs. Policies must promote the involvement of individuals with lived experience in designing, delivering, and evaluating services. Ongoing funding is vital to support capacity building, data collection and robust evaluation, ensuring continuous improvement and long-term sustainability. Currently, the work on the dashboard platform (qualityevaluation.com) emphasises automation and improved usability for real-time data use and informed decision-making. Further development should explore integrating data from local sites to provide a more comprehensive and dynamic view of service needs and outcomes.

### Future Directions

Although some localities have successfully executed this OOHC model, scaling it up is problematic due to a shortage of suitable properties. The location may be less critical, suggesting that medical respite care or care home-type facilities could serve as viable alternatives for those with higher support needs. Additional research is required to evaluate the cost-effectiveness of these varied approaches.

Our current work on the dashboard platform (qualityevaluation.com) focuses on making it more automated and user-friendly, so that data can be accessed in real time to support informed decision-making. Looking ahead, further development should aim to integrate data from local sites. This would provide a richer, more dynamic picture of service needs and outcomes, helping commissioners and providers to respond quickly and effectively.

Future research should also explore how participatory DCEs can be adapted for other marginalised populations and used as part of service evaluations. This would ensure that the voices of those who are often excluded continue to shape care models, making services more equitable, person-centred, and sustainable.

## Conclusion

This study demonstrates the value of participatory DCEs in understanding the preferences of PEH for OOHC services. The findings highlight the importance of prioritising accommodation, fostering trust, and minimising behavioural restrictions in service design. The participatory approach ensured that the research was relevant, meaningful, and impactful, empowering individuals with lived experience to shape the services that affect their lives. These insights provide actionable guidance for commissioners and service providers seeking to improve OOHC for PEH and to contribute to their journeys towards stable housing and well-being.

## Additional File

The additional file for this article can be found as follows:

10.5334/ijic.9846.s1 Supplementary Material. Appendix 1 to 4.

## References

[1] Vondeling H. Economic evaluation of integrated care: an introduction. Int J Integr Care. 2004;4:e20. Epub 2004 Mar 1. PMID: 16773144; PMCID: PMC1393259. DOI: 10.5334/ijic.95 16773144 PMC1393259

[2] Sittimart M, et al. An overview of the perspectives used in health economic evaluations. Cost Eff Resour Alloc. 2024 May 14;22(1):41. DOI: 10.1186/s12962-024-00552-1 38741138 PMC11092188

[3] Flatau P, Zaretzky M. The economics of homelessness. Palgrave Macmillan; 2008.

[4] Aldridge RW, Menezes D, Lewer D, et al. Causes of death among homeless people: a population-based cross-sectional study of linked hospitalisation and mortality data in England. Wellcome Open Res. 2019;4:49. DOI: 10.12688/wellcomeopenres.15151.1 30984881 PMC6449792

[5] Fazakerley JA, Lemos J, Wood L. ‘It’s more than just a house’: A qualitative exploration of the meaning of ‘home’ for people experiencing homelessness. Housing Stud. 2017;32(4):514–531.

[6] Ferrara L, Ardito V, Tozzi VD, Tarricone R. Economic Evaluations of Health Service Interventions Targeting Patients with Multimorbidities: A Scoping Literature Review. International Journal of Integrated Care. 2025;25(1):3. DOI: 10.5334/ijic.8623 PMC11760752 39866291

[7] Clark M, Cornes M, Whiteford M, et al., Homelessness and integrated care: an application of integrated care knowledge to understanding services for wicked issues. Journal of Integrated Care. 2022;30(1):3–19. DOI: 10.1108/JICA-03-2021-0012

[8] Clark M, Cornes M, Tinelli M, et al. Integrating homelessness support – developing a relational understanding. Journal of Integrated Care. 2025;33(1);1–17. DOI: 10.1108/JICA-05-2024-0021

[9] International Foundation for Integrated Care (IFIC). Nine pillars of integrated care [Internet]. Utrecht: IFIC; [cited 2026 Jan 14]. Available from: https://integratedcarefoundation.org/nine-pillars-of-integrated-care

[10] Jeleff M, Haider S, Schiffler T, Gil-Salmerón A, Yang L, Barreto Schuch F, Grabovac I. Cancer risk factors and access to cancer prevention services for people experiencing homelessness. Lancet Public Health. 2024 Feb;9(2):e128–e146. DOI: 10.1016/S2468-2667(23)00298-0 38307679

[11] Hoedemakers M, Karimi M, Jonker M, Tsiachristas A, Rutten-van Mölken M. Heterogeneity in preferences for outcomes of integrated care for persons with multiple chronic diseases: a latent class analysis of a discrete choice experiment. Qual Life Res. 2022 Sep;31(9):2775–2789. DOI: 10.1007/s11136-022-03147-6 35585287 PMC9356934

[12] Tinelli M. Applying discrete social experiments in social care research. Method Reviews. 2016:19. NIHR, SSCR, London, UK.

[13] Ryan M, Gerard K. Using discrete choice experiments to value health care programmes. Health Econ. 2003;12(6):431–446. DOI: 10.1093/oso/9780198527695.003.0026 14619274

[14] Lancsar S, Louviere JJ. Conducting discrete choice experiments to inform healthcare decision making: a practical guide. Pharmacoeconomics. 2008;26(8):661–677.DOI: 10.2165/00019053-200826080-00004 18620460

[15] Rocks S, Berntson D, Gil-Salmerón A, Kadu M, Ehrenberg N, Stein V, Tsiachristas A. Cost and effects of integrated care: a systematic literature review and meta-analysis. Eur J Health Econ. 2020 Nov;21(8):1211–1221. DOI: 10.1007/s10198-020-01217-5 32632820 PMC7561551

[16] Schiffler T, Kapan A, Gansterer A, Pass T, Lehner L, Gil-Salmeron A, McDermott DT, Grabovac I. Characteristics and Effectiveness of Co-Designed Mental Health Interventions in Primary Care for People Experiencing Homelessness: A Systematic Review. Int J Environ Res Public Health. 2023 Jan 3;20(1):892. DOI: 10.3390/ijerph20010892 36613214 PMC9820061

[17] Gil-Salmerón A, Carmichael C, Fragner T, Moudatsou M, Tabaki I, Cortes JB, Doñate-Martínez A, Smith L, Grabovac I. Cancer Prevention and Screening for People Experiencing Homelessness: Co-Designing the Health Navigator Model. Int J Integr Care. 2025 Nov 11;25(4):11. PMID: 41244881; PMCID: PMC12617427. DOI: 10.5334/ijic.9293 PMC12617427 41244881

[18] Abma TA, Nierse A, Widdershoven FG. Participatory health research: A practical guide. Sage; 2016.

[19] Bridges JF, et al. Conjoint analysis applications in health—a checklist: a report of the ISPOR Good Research Practices for Conjoint Analysis Task Force. Value Health. 2011;14(4):403–413. DOI: 10.1016/j.jval.2010.11.013 21669364

[20] Milne R, et al. Ethical issues in the development of readiness cohorts in Alzheimer’s disease research. J Prev Alzheimers Dis. 2017;4(2):125–131. DOI: 10.14283/jpad.2017.5 29186282

[21] Alemu MH, Mørkbak MR, Olsen SB, Jensen CL. Attending to the reasons for attribute non-attendance in choice experiments. Environ Resour Econ. 2013;54(3):333–359. DOI: 10.1007/s10640-012-9597-8

[22] Hensher DA, Rose JM, Beck MJ. Incorporating stakeholder participation in discrete choice experiments: Lessons from a study on urban mobility. Transp Res Part A Policy Pract. 2022;155:102–118.

[23] Coast J, et al. Using qualitative methods for attribute development for discrete choice experiments: issues and recommendations. Health Econ. 2012;21(6):730–741. DOI: 10.1002/hec.1739 21557381

[24] Cornes M, et al. Evaluation of the Out-of-Hospital Care Models Programme for People Experiencing Homelessness. London: NIHR Policy Research Unit in Health and Social Care Workforce, The Policy Institute, King’s College London; 2024.

[25] Rocks S, Berntson D, Gil-Salmerón A, Kadu M, Ehrenberg N, Stein V, Tsiachristas A. Cost and effects of integrated care: a systematic literature review and meta-analysis. Eur J Health Econ. 2020 Nov;21(8):1211–1221. Epub 2020 Jul 6. PMID: 32632820; PMCID: PMC7561551. DOI: 10.1007/s10198-020-01217-5 32632820 PMC7561551

[26] Schiffler T, Kapan A, Gansterer A, Pass T, Lehner L, Gil-Salmeron A, McDermott DT, Grabovac I. Characteristics and Effectiveness of Co-Designed Mental Health Interventions in Primary Care for People Experiencing Homelessness: A Systematic Review. Int J Environ Res Public Health. 2023 Jan 3;20(1):892. PMCID: PMC9820061. DOI: 10.3390/ijerph20010892 36613214 PMC9820061

[27] Gil-Salmerón A, Carmichael C, Fragner T, Moudatsou M, Tabaki I, Cortes JB, Doñate-Martínez A, Smith L, Grabovac I. Cancer Prevention and Screening for People Experiencing Homelessness: Co-Designing the Health Navigator Model. Int J Integr Care. 2025 Nov 11;25(4):11. PMID: 41244881; PMCID: PMC12617427. DOI: 10.5334/ijic.9293 PMC12617427 41244881

[28] Coast J, Al-Janabi A, Ryan M. What is the best way to use discrete choice experiments to value health care interventions? Pharmacoeconomics. 2010;28(11):999–1012.

[29] Wallerstein NB, Duran B, Oetzel J, Minkler M. Community-based participatory research for health: Advancing social justice. John Wiley & Sons; 2018.

[30] Israel GD, Schulz AJ, Parker EA, Becker AB. Review of community-based participatory research: Assessing partnership approaches to improve public health. Annu Rev Public Health. 1998;19(1):173–202. DOI: 10.1146/annurev.publhealth.19.1.173 9611617

[31] Cargo M, Mercer SL. The value of participatory research for health promotion research. Health Promot Pract. 2004;5(3):249–263.

[32] O’Brien MA, Kho ME, Detsky AS. Exploring perspectives on conducting participatory research with vulnerable populations: a qualitative study. J Gen Intern Med. 2014;29:1269–1275.

[33] Reed Johnson F, et al. Constructing experimental designs for discrete-choice experiments: report of the ISPOR Conjoint Analysis Experimental Design Good Research Practices Task Force. Value Health. 2013;16(1):3–13. DOI: 10.1016/j.jval.2012.08.2223 23337210

[34] Ride J, et al. A Reporting Checklist for Discrete Choice Experiments in Health: The DIRECT Checklist. Pharmacoeconomics. 2024 Oct;42(10):1161–1175. Epub 2024 Sep 3. PMID: 39227559; PMCID: PMC11405421. DOI: 10.1007/s40273-024-01431-6 39227559 PMC11405421

[35] Boateng GO, Neilands TB, Barnett S, Dahly DL, West BT. Best practices for developing and validating scales for health, social, and behavioral research: A primer. Front Public Health. 2018;6:149. DOI: 10.3389/fpubh.2018.00149 29942800 PMC6004510

[36] Breuer E, De Leeuw E, Kok G. Which intervention design methods are most conducive to the implementation of complex health behaviour interventions? Implement Sci. 2010;5(1):9.20205873

[37] Willis GB. Analysis of the cognitive interview in questionnaire design. Oxford: Oxford University Press; 2015.

[38] Hensher DA, Rose JM, Greene WH. Applied choice modelling. Cambridge University Press; 2015. DOI: 10.1017/CBO9781316136232

[39] Orme BK. Getting Started with Conjoint Analysis: Strategies for Product Design and Pricing Research. 2nd ed. Madison, WI: Research Publishers LLC; 2010.

[40] Cornwall A, Jewkes R. What is participatory research? Soc Sci Med. 1995;41(12):1667–1685. DOI: 10.1016/0277-9536(95)00127-S 8746866

[41] LSE. 2024. https://www.lse.ac.uk/research/research-for-the-world/society/empowering-homeless-rebuild-lives.

[42] Tsemberis S, Gulcur L, Nakae M. Housing First, consumer choice, and harm reduction for homeless individuals with a dual diagnosis. Am J Public Health. 2004 Apr;94(4):651–656. DOI: 10.2105/AJPH.94.4.651 15054020 PMC1448313

[43] Pleace N. Housing First Guide Europe. Brussels: FEANTSA; 2016.

[44] Bretherton J, Pleace N. Housing First in England: An Evaluation of Nine Services. York: University of York; 2015.

[45] Parsell C, Watts B. Charity and justice: A reflection on new forms of homelessness provision in Australia. Eur J Homelessness. 2017;11(2):65–76.

[46] Tracy DK, Lloyd LC, Shergill SS, Hanson K. Integrated care in practice: lessons from three tiers of healthcare provider and commissioner staff in two London Integrated Care Systems. BJPsych Open. 2025 Sep 15;11(5):e214. DOI: 10.1192/bjo.2025.10841 40947827 PMC12451555

[47] Corrigan O, Danielsen S, Doherty S, Lane P. Integrated care systems in England: the significance of collaborative community assets in promoting and sustaining health and wellbeing. Front Sociol. 2024 Aug 6;9:1355215. DOI: 10.3389/fsoc.2024.1355215 39165862 PMC11334352

[48] Lashley M. Engaging populations experiencing homelessness in nursing research: Barriers and best practices. J Radiol Nurs. 2024;43(1):15–20. DOI: 10.1016/j.jradnu.2023.07.001

[49] Jagosh J, et al. Uncovering the benefits of participatory research: implications of a realist review for health research and practice. Milbank Q. 2012;90(2):311–346. DOI: 10.1111/j.1468-0009.2012.00665.x 22709390 PMC3460206

[50] Fawcett SB, et al. A conceptual model of community empowerment: Implications for prevention and community psychology. Am J Community Psychol. 1995;23(5):677–706. DOI: 10.1007/BF02506987 8851345

[51] Viswanathan M, et al. Community-based participatory research: assessing the evidence. Evid Rep Technol Assess (Summ). 2004;(99):1–8. DOI: 10.1037/e439622005-001 PMC4780908 15460504

[52] Padgett DK, Stanhope V, Henwood BF, Stefancic A. Substance use outcomes among homeless clients with serious mental illness: comparing Housing First with Treatment First programs. Community Ment Health J. 2011;47(2):227–232. DOI: 10.1007/s10597-009-9283-7 20063061 PMC2916946

[53] Dayton E, Crowe KE, Simmelink J, Cronley C. Therapeutic relationships: Essential for successful interventions with homeless individuals. J Community Psychol. 2011;39(2):181–193.

[54] Lathrop B. Nursing leadership in addressing the social determinants of health. Policy Polit Nurs Pract. 2013;14(1):41–47. DOI: 10.1177/1527154413489887 23793135

