# Supplementary material for: Measuring What Matters: Using Participatory Choice Modelling to Understand Preferences for Homelessness Integrated Care
Source: Int J Integr Care. 2026 Aug 5;26(1):6. doi: 10.5334/ijic.9846 (PMC13450268; doi:10.5334/ijic.9846)

Supplementary material

Appendix 1: Service options considered in the choice tasks, their characteristics and levels

	**Service A / B (Specialist Intermediate Care)**	**Returning to rough sleeping with visits to A&E**
**(characteristics)**	**(levels)**	**(levels)**
**Location of care**	You are in a **care home environment.**<br>You are in a **hospital environment.**<br>You are in your **own flat/house.**<br>You are in **shared flat/house.**<br>You are in a **hotel** environment.<br>You are in a **hostel** environment.	You are **rough sleeping.**
**The professional who delivers most of your care (as part of a team with other professionals)**	A **nurse** delivers most of your care.<br>A **social worker** delivers most of your care.<br>A **housing support worker** delivers most of your care.<br>A **peer navigator** delivers most of your care.<br>A **mental health support worker** delivers most of your care.<br>A **drug and alcohol support worker** delivers most of your care.	A **nurse** delivers most of your care.<br>A **social worker** delivers most of your care.<br>A **housing support worker** delivers most of your care.<br>A **peer navigator** delivers most of your care.<br>A **mental health support worker** delivers most of your care.<br>A **drug and alcohol support worker** delivers most of your care.
**How often you receive a visit from one of the professionals who provide your care**	You are seen **1 to 2 times per week.**<br>You are seen **3 to 4 times per week.**<br>You are seen **5 to 6 times per week.**<br>You are seen more than **7 times per week.**	You are seen **1 to 2 times per week.**<br>You are seen **3 to 4 times per week.**<br>You are seen **5 to 6 times per week.**<br>You are seen more than **7 times per week.**
**How long for you receive care after hospital**	Your care is provided for **4-5 weeks.**<br>Your care is provided for **6-7 weeks.**<br>Your care is provided for **8-9 weeks.**<br>Your care is provided for **10-12 weeks.**	Your care is provided for **4-5 weeks.**<br>Your care is provided for **6-7 weeks.**<br>Your care is provided for **8-9 weeks.**<br>Your care is provided for **10-12 weeks.**

	Service A / B (Specialist Intermediate Care)	Returning to rough sleeping with visits to A&E
(characteristics)	(levels)	(levels)
**Rules about behaviour where you live**	There are **no rules** about your behaviour.<br>There are **less strict rules** about your behaviour.<br>There are **strict rules** about your behaviour.<br>There are **very strict rules** about your behaviour.	There are **no rules** about your behaviour.

Note: Participants were asked to choose between two care options: Service A/B, representing alternative specialist intermediate care models offering structured support following hospital discharge, and Rough Sleeping, representing a return to the street with the possibility of attending A&E. Each option was described using a set of care characteristics (attributes), with distinct levels specified for Service A/B and for Rough Sleeping.

Appendix 2: Experimental design and analysis (Methods)

The Discrete Choice Experiment (DCE) was designed using three unlabelled scenarios. The structure included one attribute with seven levels, one with six levels, and three with four levels. Constraints were applied, with one alternative always fixed as the "rough sleeping" scenario. The alternative scenario included all possible attribute levels, except for one attribute (location of care), where only six of the seven levels were applicable. The remaining level (sleeping rough) was assigned to the fixed scenario.

This design resulted in a total of 2304 potential choice sets. A design optimised for the Multinomial Logit (MNL) model, based on D-efficiency criteria, was generated using Ngene software (https://www.choice-metrics.com/), producing 12 choice sets. Figure 1 provides an example. The scenarios were reviewed to ensure logical consistency, with all attribute levels found to be applicable and coherent.

Given evidence suggesting that five to six choice sets are more suitable for individuals with mild cognitive impairment (Milte et al., 2014), the survey was divided into three versions. Each version included four choice sets plus one consistency test question, totalling five questions. The consistency test presented a dominant choice set, in which one alternative (specialist intermediate care) contained only the most favourable attribute levels, while the other (rough sleeping scenario) included the least favourable levels. Preferred and non-preferred levels were identified in advance through a combination of literature review, expert consultation, and findings from earlier qualitative work with people with lived experience of homelessness. This approach ensured that the dominant choice set was constructed using attribute levels consistently considered beneficial or undesirable in this context. The inclusion of this set followed best practice for evaluating response reliability in discrete choice experiments (Ryan et al., 2005).

To ensure a balanced representation of attribute levels across the versions, an experimental design technique was applied. This approach aimed to optimise participant engagement and comprehension while maintaining the survey’s validity. Further details on blocking design to balance attribute levels can be found in Reed Johnson et al. (2013).

Regression Analysis and data utilisation:

A DCE was conducted to analyse individual decision-making. The conditional logit model, based on McFadden’s (1973) framework, was applied following Random Utility Theory, which assumes that an individual’s choice is influenced by both an explainable (fixed) component and an unexplainable (random) component.

Mixed Logit Model: This more advanced model relaxes some of the assumptions of the CLM, allowing for heterogeneity in preferences across individuals. We tested the following random parameters (one at a time, keeping the others fixed): Location of Care (Normal Distribution); Professional Delivering Care (Normal Distribution); Frequency of Visits (Normal Distribution); Duration of Care (Triangular Distribution); and Rules About Behaviour (Uniform Distribution). However, model fit comparisons based on log-likelihood, Akaike Information Criterion (AIC), and Bayesian Information Criterion (BIC) indicated that the Conditional Logit Model provided a better fit compared to any alternative model.

The results presented in this paper are based on the conditional logit model, as it effectively captures the relationship between alternative-specific attributes and choice behaviour.

Further details on the logit models are available upon request.

Direction of preferences and importance of characteristics:

The analysis focused on the direction of preferences within the intermediate care service characteristics. A positive coefficient signifies that an increase in the variable corresponds to an increased likelihood of choosing the associated characteristic. This provides a clear understanding of the factors that positively influence participants' choices.

The importance of intermediate care characteristics was assessed using the p-value linked to each coefficient. A low p-value (typically below 0.10) indicates the statistical significance of the variable's impact on choices. The size of the coefficients was also considered; larger coefficients denote a more substantial influence on choices, highlighting the relative importance of specific attributes.

Relative importance of intermediate care characteristics:

To determine the relative importance of each attribute level in decisions regarding intermediate care, we calculated the marginal probabilities associated with each attribute level. These probabilities were derived from the estimated utility coefficients obtained from the Multinomial Logit model. The utility coefficients represent the strength and direction of preference for each attribute level (Friedel, et al 2022).

The marginal probability for each alternative was calculated using the following logit model formula:

$$P(y=j) = \frac{e^{U_j}}{\sum_{k=1}^{J} e^{U_k}}$$

Where P(y=j) is the probability of choosing alternative j, and Uj is the utility of alternative j, which is the sum of the attribute coefficients multiplied by their respective levels.

To assess the relative importance of each attribute, we compared the change in probabilities associated with changes in each attribute level. The change in probability for each attribute was calculated by comparing the probabilities of choosing an alternative before and after altering the attribute level.

The relative importance of each attribute was then quantified by calculating the difference in probability between the highest and lowest levels of each attribute. This difference, or range of probabilities, was used to determine the impact of each attribute on the decision-making process.

To express the relative importance of each attribute in percentage terms, we used the following formula:

$$\text{Relative Importance of Attribute} = \frac{\Delta P_{\text{attribute}}}{\sum_{i=1}^{N} \Delta P_i}$$

Where $\Delta P_{\text{attribute}}$ is the change in probability for each level of the attribute, and $\sum_{i=1}^{N} \Delta P_i$ represents the total change in probability across all attributes and levels.

This methodology allowed for the identification of which attributes had the greatest influence on the choices made by participants, providing insights into the factors that are most likely to drive decisions regarding intermediate care.

Frequency of preferences:

The frequency of preferences for each service characteristic was analysed, with a breakdown provided for each attribute level. For example, preferences for accommodation type were assessed, showing a breakdown according to the following categories: care home, hospital, own flat/house, shared flat/house, hotel, hostel, or rough sleeping. This analysis provided insights into the most and least preferred accommodation settings (alongside breakdowns for other characteristics), helping to identify key factors influencing service uptake and engagement.

Probability uptake models:

The probabilities derived from DCE data were used for predictive modelling of uptake rates or preferences for different models of care or services. Comparing different scenarios provided insights into how preferences may vary between a baseline option (e.g., rough sleeping) and alternative services (each representing different specialist hospital discharge schemes). This approach can identify optimal service provision that meets stakeholder requirements and has the best chance of long-term sustainability in preference to returning to rough sleeping.

Subgroup analysis:

Subgroup analyses were conducted based on age, gender, location, and type of service received to explore variations in choice frequency, regression analysis results, and service provision uptake.

Choice frequency for each attribute characteristic was analysed for the entire cohort and subgroups based on age, gender, location, and type of service received. Differences across subgroups were tested using chi-square tests for categorical variables.

For the regression analysis, the smaller sample size precluded reliable subgroup analyses; therefore, regression analysis was conducted for the entire cohort only.

Theoretical validity of responses and consistency test:

Theoretical validity of responses was assessed by examining the signs of attribute coefficients. It was expected that respondents would prefer:

- Alternative locations to rough sleeping, with a private flat or house being the most preferred option (i.e., positive β coefficients for these attributes).
- Care provided by professionals other than nurses (i.e., positive β coefficients for these attributes).
- A higher frequency of care (i.e., positive β coefficients).
- A longer duration of care (i.e., positive β coefficients).

- Less rules (i.e. negative β coefficients).

To assess response consistency, a dominant choice set was included, where one service contained all the most preferred attribute levels while the other included the least preferred levels (Ryan et al., 2005).

References

Friedel JE, Foreman AM, Wirth O. (2022). An introduction to "discrete choice experiments" for behavior analysts. Behav Processes, 198, 104628.

McFadden, D. (1973). Conditional Logit Analysis of Qualitative Choice Behavior. In: Zarembka, P. (ed.) Frontiers in Econometrics. Academic Press, New York, pp. 105-142.

Milte, R., Ratcliffe, J., & Larkins, S. (2014). The optimal number of choice sets in a discrete choice experiment for individuals with cognitive impairments: Evidence from a study on health care preferences. Health Economics, 23(4), 392-402.

Ryan, M., Gerard, K., & Amaya-Amaya, M. (2005). Using Discrete Choice Experiments to Value Health and Health Care. Springer, Dordrecht.

Reed Johnson, F., Lancsar, E., & Marshall, D. (2013). Designing discrete choice experiments to estimate preferences for health and healthcare. BMJ, 347, f4505.

Appendix 3: Regression analysis results (Preferred model used for analysis: conditional logit)

Table 3.1: Main analysis (n=106)

Service characteristics (attributes)	Levels	Beta val	SE	P.val
**Location of care**	You are **rough sleeping**	-4.71	0.59	<0.01
	You are in a **hospital environment.**	-1.057	0.463	0.02
	You are in your **own flat/house.**	1.02	0.5	<0.01
	You are in **shared flat/house.**	-1.51	0.37	<0.01
	You are in a **hotel** environment.	-0.19	0.31	<0.01
	You are in a **hostel** environment.	-0.192	0.5	<0.01
**(compared with rough sleeping)**				
**The professional who delivers most of your care (as part of a team with other professionals)**	A **nurse** delivers most of your care.	-0.141	0.379	0.71
	A **housing support worker** delivers most of your care.	0.116	0.371	0.75
	A **peer navigator** delivers most of your care.	-0.240	0.422	0.57
	A **mental health support worker** delivers most of your care.	-0.717	0.422	0.09
	A **social worker** delivers most of your care	-0.221	0.424	0.60
**(compared with drug and alcohol support worker)**				
**How often you receive a visit from one of the professionals who provide your care**	**Frequency** (Increase from 1-2 times per week to more than 7 times per week).	-0.131	0.126	0.30
**How long for you receive care after hospital**	**Duration** (Increases from 4-5 weeks to 10-12 weeks)	0.0482	0.095	0.61
**Rules about behaviour where you live**	**Strictness of the rules** (Increases from no rules to very strict rules)	-0.383	0.145	<0.01

Appendix 4: Frequency of choice and breakdown according to sociodemographic characteristics, location and experience of care.

This dashboard presents a detailed overview of frequency breakdown of service preferences by location of care. The demographic breakdown: each graph presents a separate subgroup analysis according to gender (male/female) and age. We also considered breakdown according to location (in and outside London) as well as type of services (pathway 1 or pathway 2).

Figure 4.1: Location of care

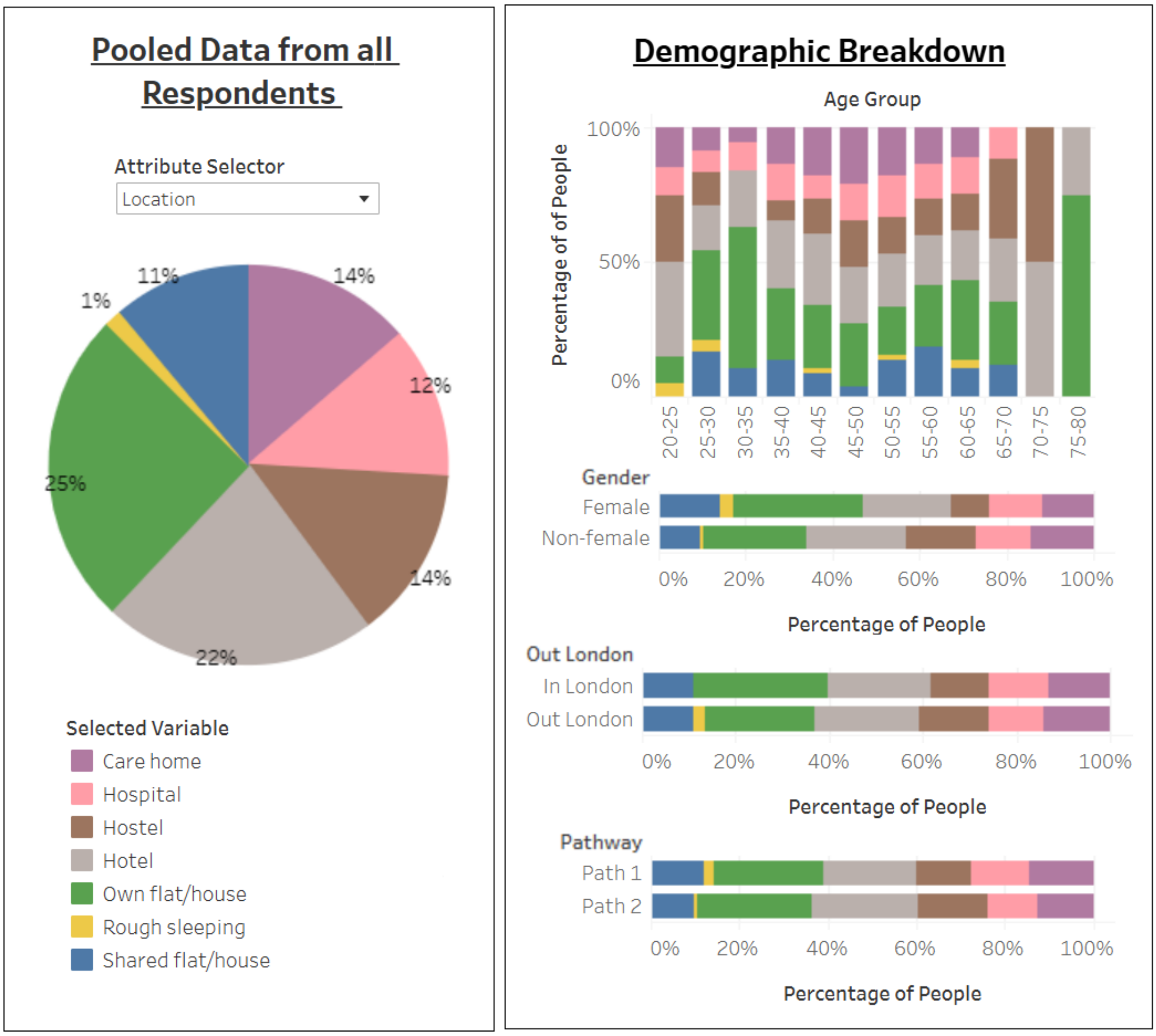

Figure 4.2: Rules about behaviour

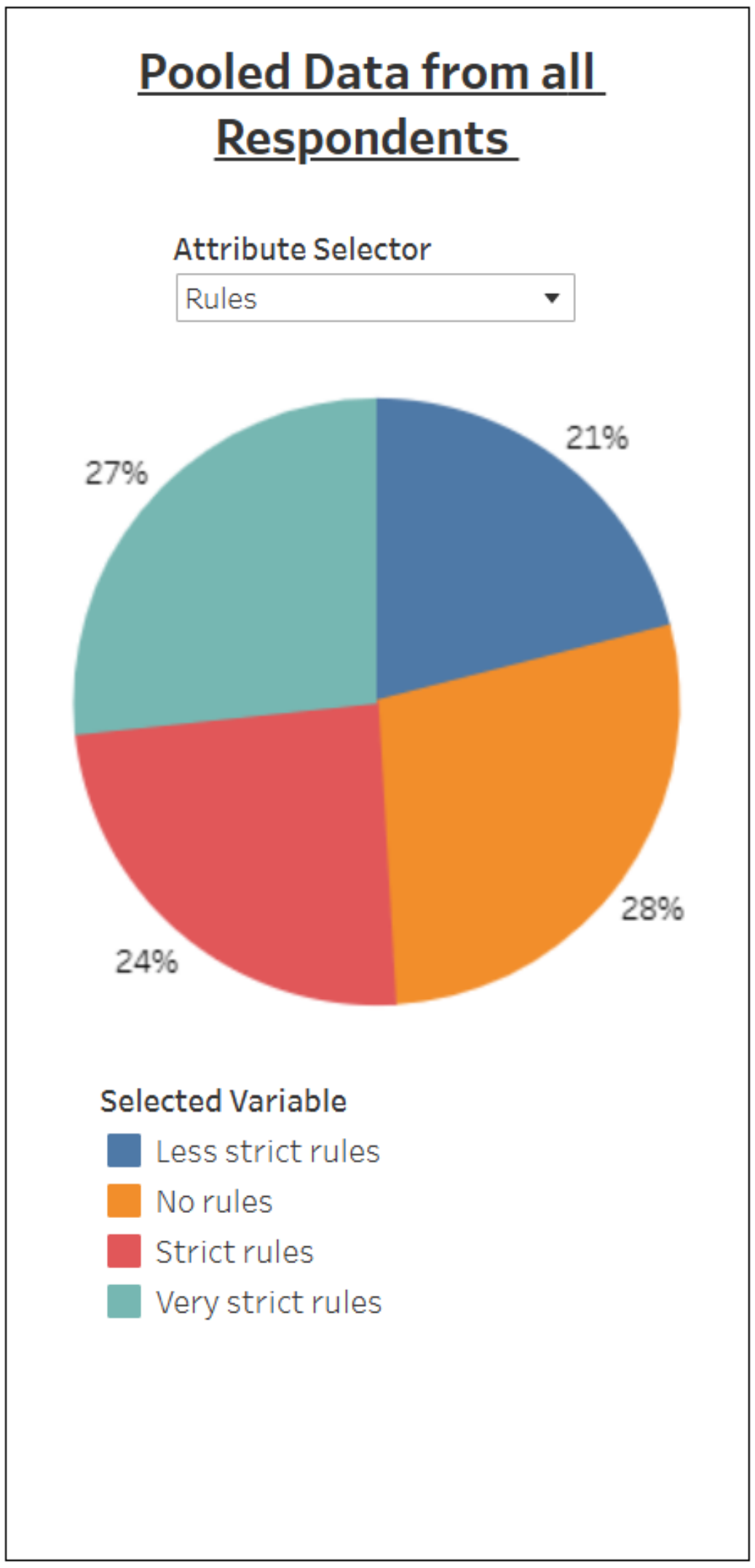

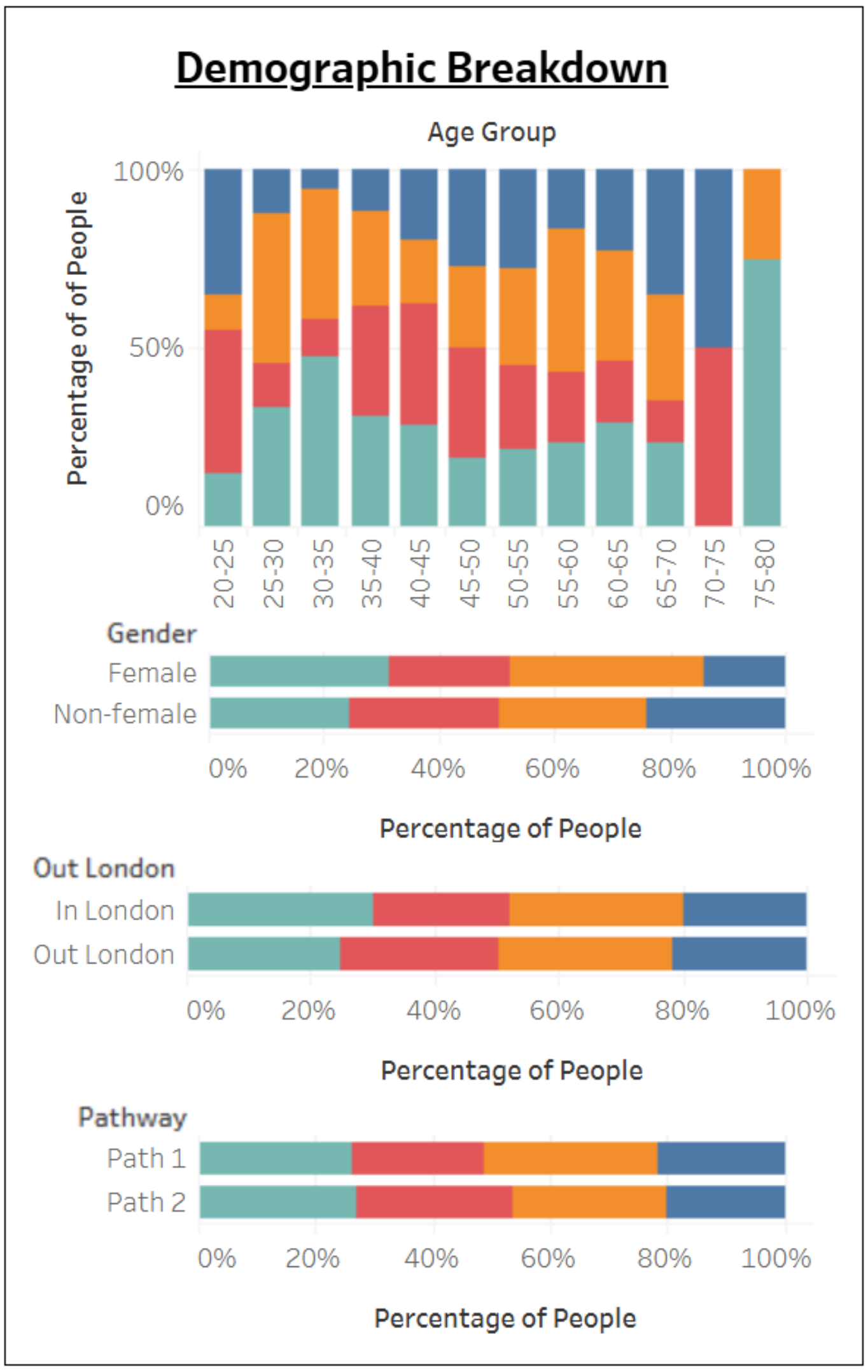

Figure 4.3: Primary carer

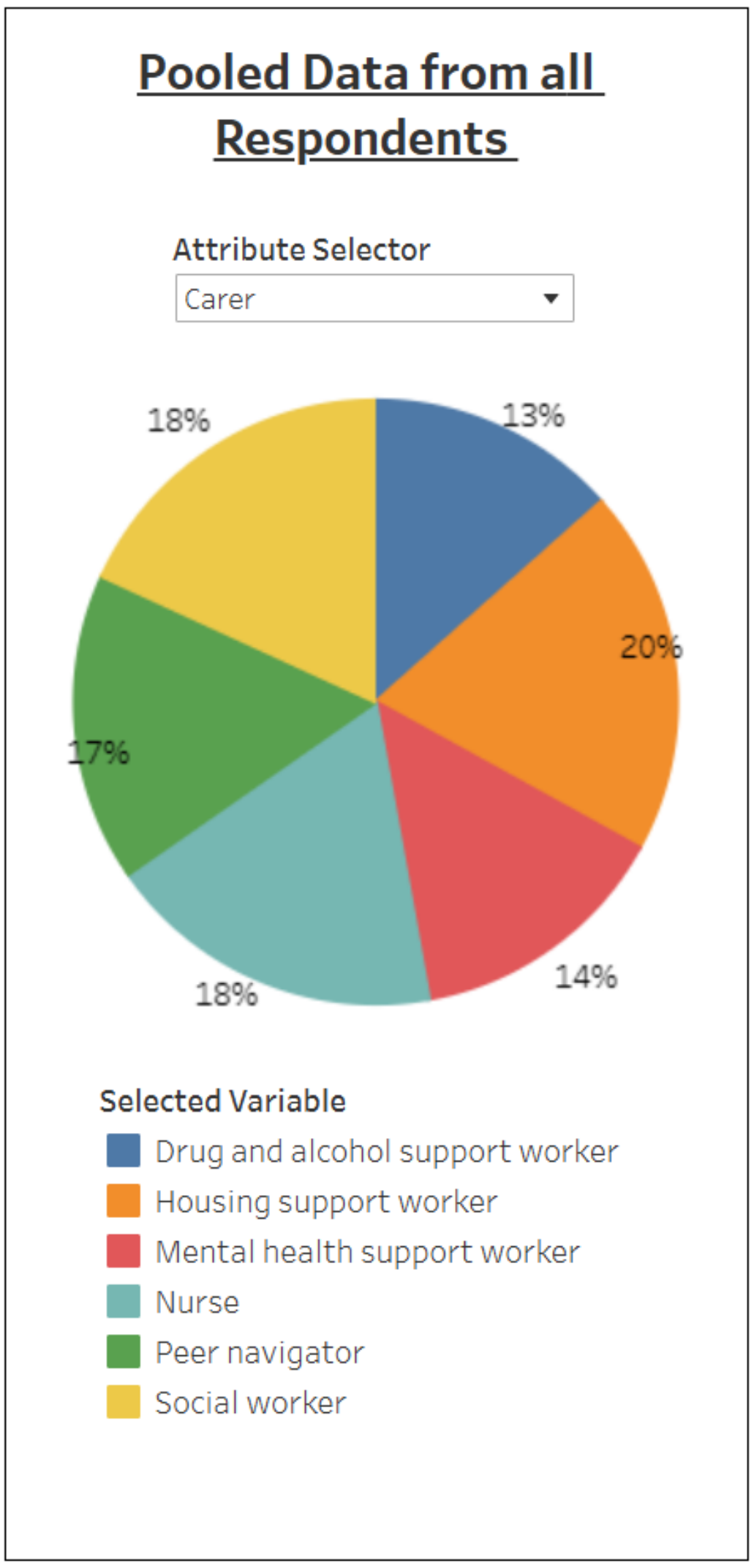

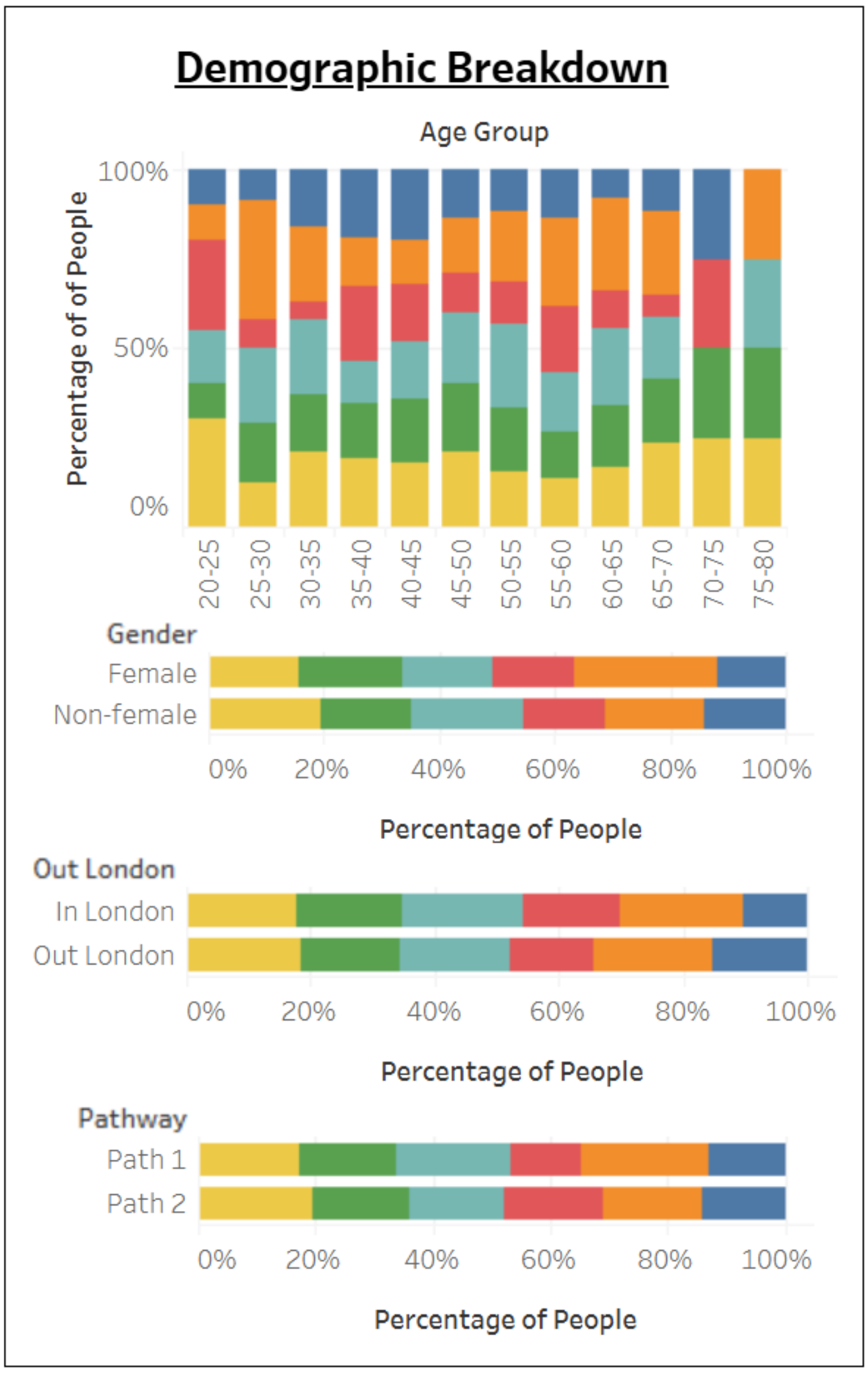

Figure 4.4: Duration of care

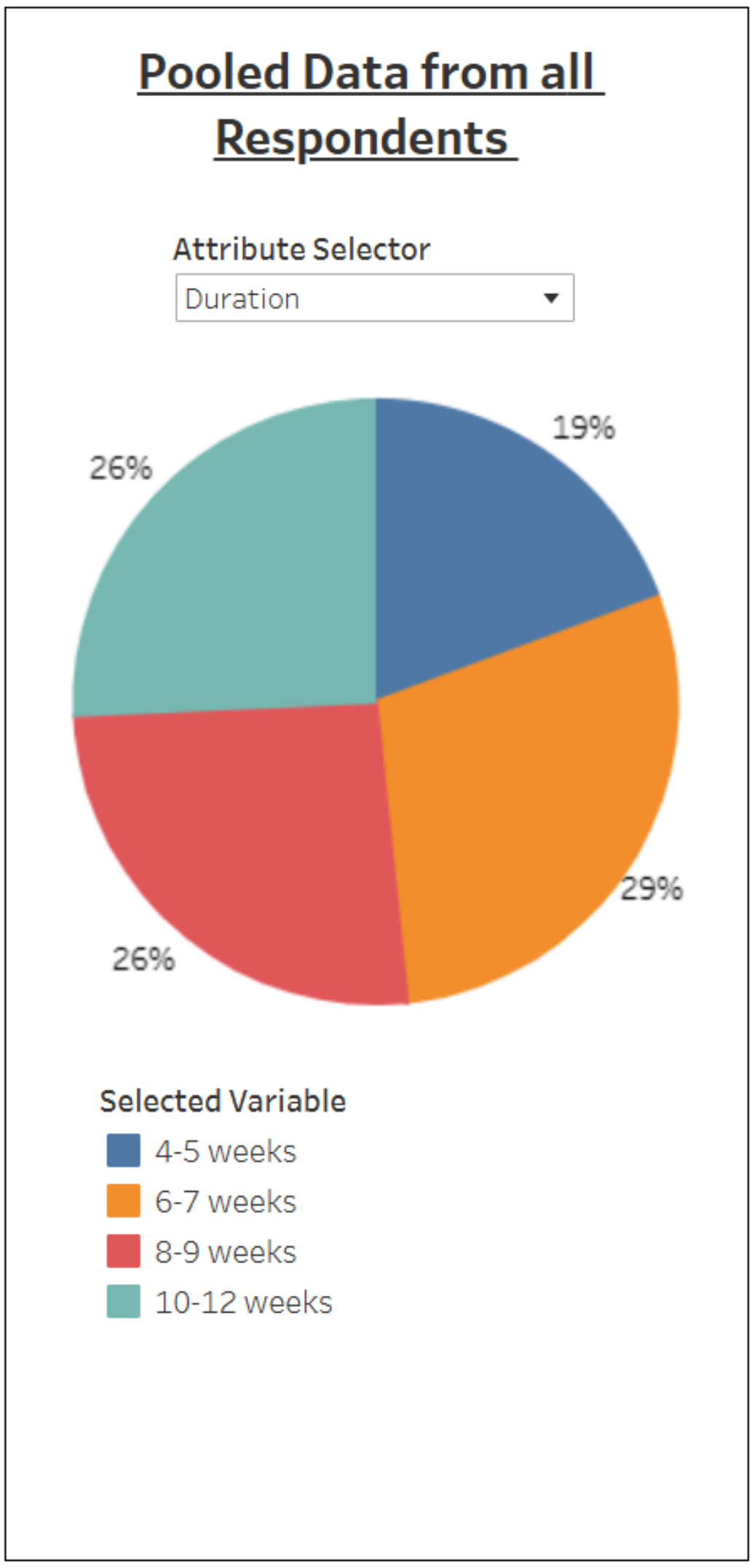

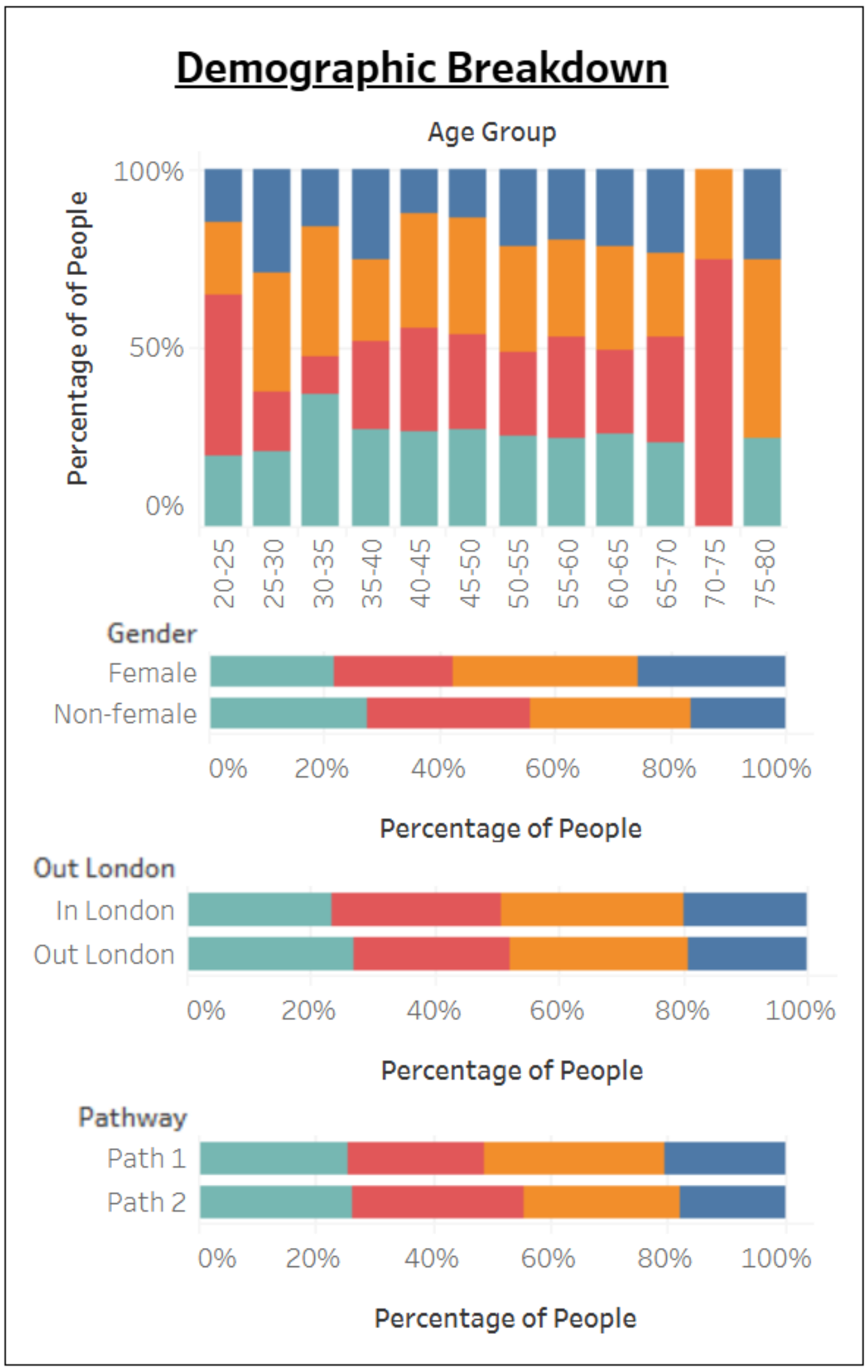

Figure 4.5: Frequency of care

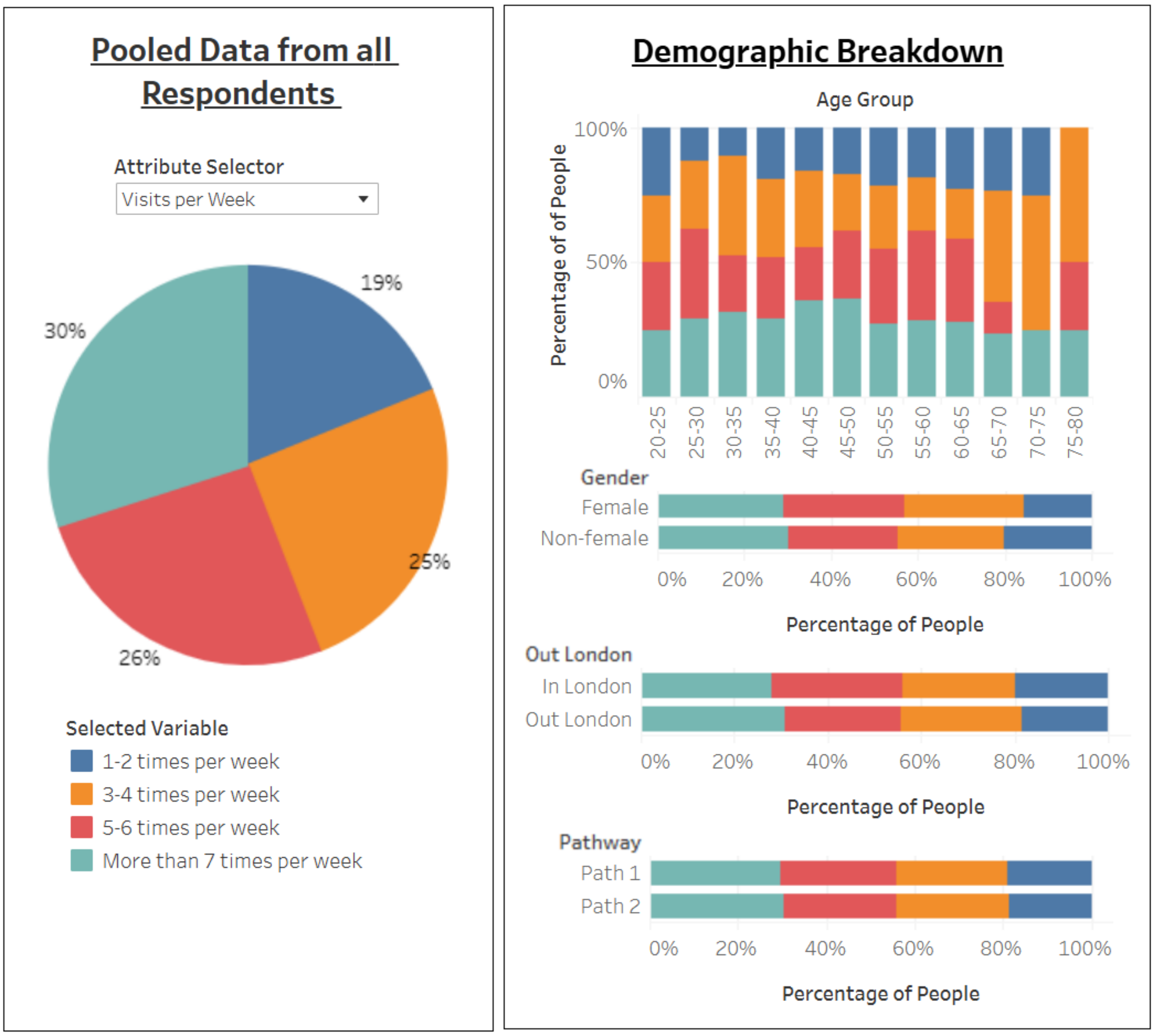

Supplement: Supplementary file 1 [file ijic-26-3-9846-s1.pdf]
